# A highly variable habitat selection in moose across diel and seasonal scales

**DOI:** 10.1186/s40462-024-00508-3

**Published:** 2024-10-09

**Authors:** Tomasz Borowik, Rafał Kowalczyk, Mirosław Ratkiewicz, Weronika Maślanko, Norbert Duda, Michał Żmihorski

**Affiliations:** 1 Mammal Research Institute, Polish Academy of Sciences, Stoczek 1, Białowieża, 17-230 Poland; 2 Faculty of Biology, University of Białystok, Ciołkowskiego 1 J, Białystok, 15-245 Poland; 3 Department of Animal Ethology and Wildlife Management, University of Life Sciences in Lublin, Akademicka 13, Lublin, 20-950 Poland; 4 Zespół Szkół Ogólnokształcących, Nr 2 w Białymstoku, Narewska 11, Białystok, 15-840 Poland

**Keywords:** Temporal scales, Habitat selection, Step-selection function, Generalized additive models, Moose

## Abstract

**Purpose:**

Habitat selection in animals is a hierarchal process that operates across multiple temporal and spatial scales, adapting to changes in environmental conditions, human disturbances, and predation risks. Despite its significance, previous research often oversimplifies temporal dynamics by categorizing them into broad seasonal and diel patterns, overlooking the continuous nature of temporal variability and habitat specificity.

**Methods:**

We investigated the temporal patterns in habitat selection of moose (*Alces alces*) in highly heterogenous landscapes at the southwestern edge of their European range using step-selection functions. Utilizing over 700,000 GPS locations from 34 adult moose, we aimed to assess seasonal and diel patterns in their selectivity for both natural and human-related habitats.

**Results:**

Our findings revealed significant overall temporal variation in moose habitat selection at both seasonal and diel scales. Moose selectivity toward different habitats showed low repeatability over time, with 35% of cases displaying negative correlation between selectivity in different time windows. Diel changes were more pronounced, showing 5.6-fold difference in cumulative selectivity, compared to 1.4-fold difference in seasonal dynamics. Notably, moose exhibited lower selectivity during nighttime hours throughout the year compared to daytime hours. The study also highlighted distinct habitat selection patterns across different habitat types: natural habitats (deciduous forests, coniferous forests, wetlands) exhibited pronounced seasonal variation, while anthropogenic habitats (grasslands, arable land, roads and settlements) showed more diel variability. Moose generally avoided human-related habitats during daytime hours, but their preferences during nighttime varied depending on the habitat type and time of year.

**Conclusion:**

This research advances our understanding of the complex temporal patterns in habitat selection by large herbivores and underscores the importance of considering temporal dynamics in habitat selection modelling.

## Background

To maximize fitness, animals optimize their occupancy patterns through habitat selection, leading to the disproportionate use of certain habitat types relative to their availability [1, 2]. This occurs because habitats are heterogenous, with the various resources necessary for survival and reproduction being spatially dispersed. Habitat selection involves trade-offs, as animals must weigh the costs and benefits associated with resource quality and quantity on one hand, and factors such as locomotor costs, predation risk, competition, human disturbance, or weather conditions on the other [3, 4]. In some cases, habitats that provide high quality forage may also pose the highest risk due to elevated predation threats or human-related dangers [5–7].

In ecological studies of habitat selection in animals, substantial knowledge has been accumulated regarding the spatial context of habitat selection, including the effects of different spatial scales – from species distribution range to the ranges of specific individuals – on the selection of various resources by animals. However, within this framework, the temporal aspects of habitat selection have received considerably less attention (but see recent studies: [8–11]), despite their potential importance, which can sometimes override the significance of spatial scales [12–14]. Studying habitat selection at different temporal scales helps ecologists to understand various aspects of animal behaviour, depending on research questions. Fine-scale habitat selection (minute, hour) provides insights into immediate behavioural responses to environmental stimuli, such as foraging efficiency, social interaction, and avoidance of human or predator [15–19]. Selection at the day/night scale helps us to understand behaviours related to circadian activity patterns, while broad-scale selection, sheds light on seasonal changes in habitat use and seasonal movement patterns. For example, red deer (*Cervus elaphus*) and roe deer (*Capreoulus capreolus*) exhibited clear seasonal variation in habitat selection that corresponds the seasonal dynamics in vegetation phenology [13, 20]. Finally, tracking interannual changes in habitat selection allows researchers to identify long-term trends and shifts in habitat use, which can be critical for predicting the ecological consequences of climate change. Importantly, habitat selection at different temporal scales is not independent; the diel pattern of habitat selection may change across seasons. For instance, red deer on a military training area in Bavaria (Germany) preferred closed habitats during the day only in summer [10].

Understanding habitat selection at various temporal scales is essential for developing targeted and effective conservation and management strategies that ensure the long-term survival and health of wildlife populations. For example, conservationists can use the knowledge on habitat selection in species of conservation concern to design or evaluate protected areas and ecological corridors that accommodate both the short- and long-term needs of these species. This understanding can also inform strategies to help species adapt to changing climate. Additionally, this knowledge is valuable for developing adaptive management strategies that allow for timely actions to prevent potential human-wildlife conflicts or to counteract environmental changes [21–23].

The selection of an appropriate temporal scale in habitat selection analyses is crucial, as an inadequate scale can result in misleading conclusions. Ecologists commonly use broad classifications, such as dividing year into cold and warm periods or into four seasons (spring, summer, autumn, winter), and the day into day and night or three parts (daytime, twilight and nighttime hours) [9, 10, 24]. However, this approach may miss significant temporal patterns in habitat selection that may occur on finer scales, such as monthly, weekly, daily, or even hourly variation. For example, during summer, the declining quality of forage due to senescence may restrict habitat attractiveness to specific periods within the season [25, 26]. As a result, habitat selection coefficients in ecological models, which often average selection over longer periods, may underestimate the strength of habitat selection. Additionally, temporal variation in habitat selection can differ between habitats, as each habitat’s benefits and costs can fluctuate on different temporal scales. One habitat might show seasonal variations in selection strength, while another might vary more on a diel scale. Therefore, applying the same temporal resolution across different habitats could obscure important ecological patterns.

In our study, we aimed to demonstrate that large herbivores, specifically moose (*Alces alces*), select habitats at varying temporal scales that can differ significantly among habitat types. We also sought to emphasize the benefits of using time as a continuous variable to represent habitat selection patterns, rather than relying on predefined temporal scale classes. To explore these patterns, we analysed over 700,000 locations from 34 GPS-tracked moose in Eastern Poland, estimating habitat selection separately for seven distinct habitat types. We selected moose as the model species due to their high selectivity (‘concentrate selectors’ according to [27] and a strict browser and obligatory non-grazer sensu [28]) and clear temporal variation in behaviour within this heterogenous study area, observable both at seasonal and diel scales [19, 29–31]. We employed step-selection functions at a fine spatio-temporal scale, which allowed to account for positional autocorrelation [32–34]. We hypothesized that habitat selection would exhibit significant temporal variation, both diel and seasonal. Specifically, we expected that natural habitats (forests, wetlands) would show the greatest seasonal differences due to variations in their structure, vegetation biomass, wetness, etc. Conversely, we anticipated that human-altered habitats (arable land, grasslands, settlements, roads) would be more influenced by diel variation, primarily due to human disturbances, which are more prevalent during the day. Furthermore, we proposed that habitat selection would show an interactive effect of seasonal and diel scales, as the diel pattern of the habitat selection by moose may vary across seasons. This expectation is based on the varying risks associated with human activities throughout the year, such as hunting, timber logging, agriculture, and tourism. Finally, given the temporal variability in factors driving habitat selection (e.g., human activity, plant phenology), we anticipated that the overall strength of habitat selection by moose would not be uniform. Instead, we expected to identify specific periods, such as parts of the year or times of day, during which moose would exhibit either strong or limited habitat selection.

## Materials and methods

### Study area

**Fig. 1:**
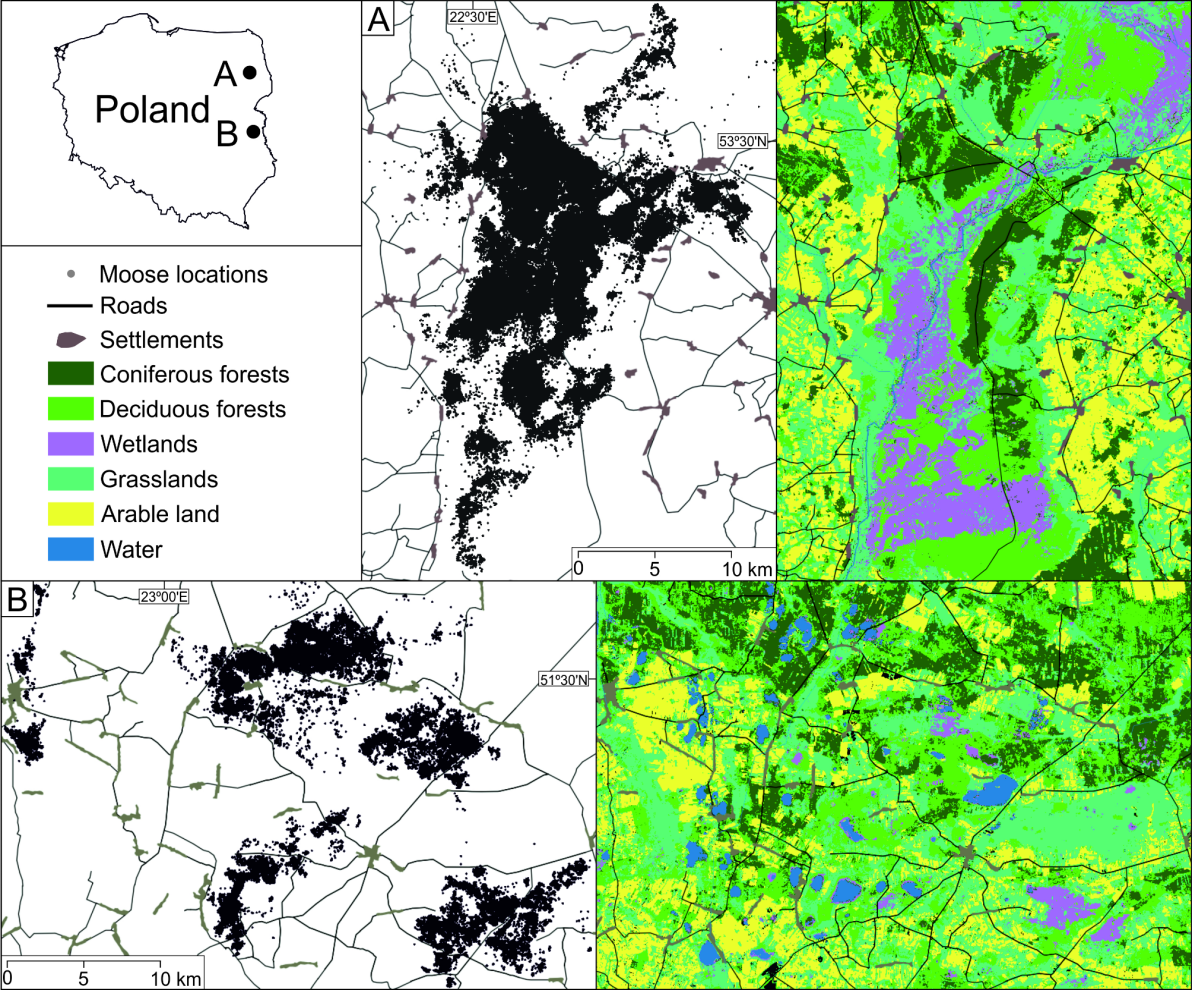
Distribution of the Biebrza (**A**) and Polesie (**B**) study sites and moose locations in Eastern Poland during GPS-tracking from 2012 to 2018

The study area included two sites in Eastern Poland (Fig. 1). The northern study site encompasses Biebrza National Park and the surrounding forest districts, collectively referred as Biebrza. This site lies within the Biebrza river valley, a peat basin 12–15 km wide, bordered by uplands 10–20 m high, predominantly covered by coniferous and mixed forests. The river valley is characterized by sedge, sedge-moss, and reed communities, while the forests are primarily composed of black alder (*Alnus glutinosa*), downy birch (*Betula pubescens*), and Scots pine (*Pinus sylvestris)*. The significant proportion of the valley is covered by birch-willow scrub. Additionally, large areas of the valley are maintained through mowing and deforestation to manage natural succession. The southern study site is lcoated in the West Polesie Biosphere Reserve, which includes Polesie National Park (Polesie). This landscape is dominated by marshes containing fen plant communities, with forests primarily composed of Scots pine, black alder, and downy birch. Both study sites are adjacent to a mosaic of grasslands used as meadows or pastures, along with arable land cultivated for cereals and rapeseed (*Brassica napus*) [35–37].

Both study sites are home to four species of ungulates: moose, red deer, roe deer, and wild boar (*Sus scrofa*), along with their predator, the wolf (*Canis lupus*) [38, 39]. In Poland, moose have been under a hunting ban since 2001 due to significant population decline caused by overharvesting [40]. Other ungulates, however, are hunted outside the boundaries of national parks [41]. Moose movement strategies differ between the two study sites, primarily due to variations in habitat patchiness. In Biebrza, where forests and marshes are less fragmented, moose tend to migrate (short distance migrations). Conversely, in the more fragmented landscape of Polesie, most moose exhibit resident behavior [29].

The climate in the study area exibits both Atlantic and continental influences. The growing season lasts approximately 200 days on average, while snow cover persists for 110–120 days, with an average maximum snow depth of 25–30 cm. July is the warmest month (Biebrza study site: mean = 18.4 ºC, range: 2.1–33.7 ºC; Polesie study site: mean = 19.7 ºC, range: 7.5–34.1 ºC), while January is the coldest (Biebrza study site: mean = -3.9 ºC, range: -23.3–8.1 ºC; Polesie study site: mean = -2.9 ºC, range: -21.5–10.9 ºC). Annual precipitation is 550 mm, with summer rains accounting for 40% of the yearly total [42, 43].

### Movement data

We used 715,278 GPS locations from 34 adult moose (aged ≥ 2 years) collected from the Biebrza (2012–2017; 13 females and 11 males: 554,164 locations) and Polesie (2013–2018; 9 females and 1 male: 161,114 locations) study sites. The moose were immobilized using etorphine [44] and fitted with Ecotone Telemetry GPS-GSM collars. Collaring was conducted during winter (January-March). No adverse effects of anaesthesia or collaring on moose behaviour were observed. The collars were programmed to record the animal’s positions every hour. We screened the GPS data for positional outliers (location errors) and removed them from the final dataset. A location was classified as an outlier if the step length exceeded 15 km within a 1-h interval.

### Step selection function and statistical analyses

We employed a step-selection function framework to assess moose habitat selection. The movement paths of individual moose were computed using “amt” package [45], and regularized by retaining only locations separated by 60 min (± 15 min), resulting in distinct bursts within each individual’s movement track. To ensure calculation of turning angles between consecutive locations, bursts with fewer than three locations were removed, yielding a dataset of 660,246 locations. Next, for each individual and each step within a burst, we generated one random step (location) by drawing random step lengths and turning angles from gamma and von Mises distributions, respectively. These distributions were parameterized using observed step lengths and turning angles, resulting in the generation of 660,246 random locations.

**Fig. 2:**
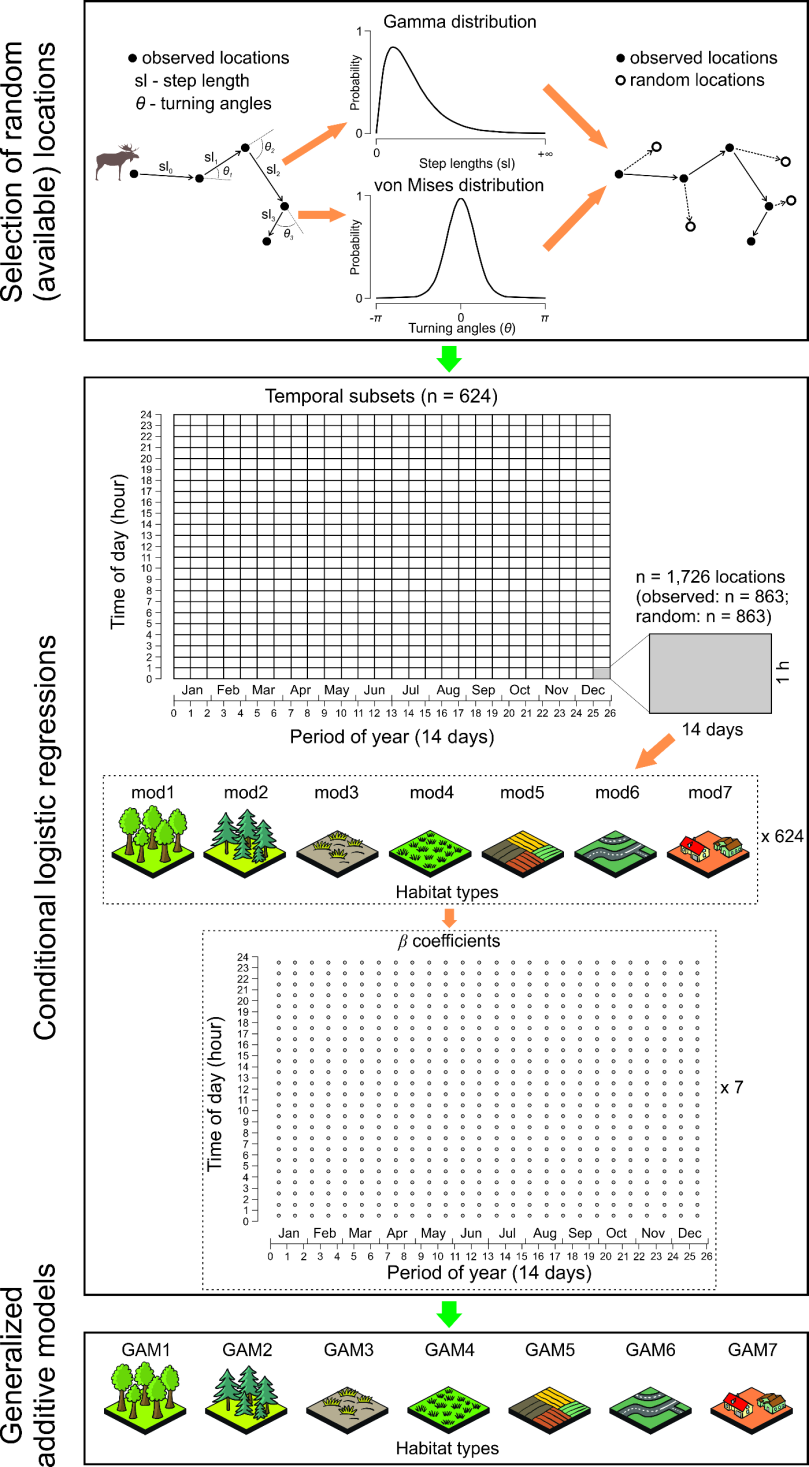
Flowchart of step-selection function and statistical analyses on temporal pattern of habitat selection of moose in Eastern Poland

Then, we divided the final database, which included both observed and random locations, into temporal subsets. Each subset represented a 14-day period and 1-hour interval, starting from the beginning of the year and midnight. For example, the first subset included data collected between January 1st and January 14th, from 00:00 to 01:00 h. We chose 14-day periods to maintain a relatively similar number of daily (24) and seasonal (26) periods. This process resulted in 624 subsets (26 14-day periods x 24 1-hour intervals), with each subset containing between 1,726 and 2,552 locations (both observed and random) (Fig. 2). To ensure comparability among the temporal subset, we standardized the number of locations in all 624 subsets by randomly selecting 1,726 locations (863 pairs) from each subset. This standardization process resulted in 624 temporal subsets, each containing 1,726 locations, totalling 538,512 observed and 538,512 random locations used for habitat selection modelling.

We measured habitat variables at the endpoint of each step for both observed and random locations across all 624 temporal subsets. These locations were intersected with a habitat map (2020; 10 × 10 m resolution) that classified nine habitat types: deciduous forests, coniferous forests, wetlands, grasslands, arable land, anthropogenic areas, moorland, natural areas deprived of vegetation, water (Polish Space Agency) [46] (Fig. 2). Due to infrequent intersection of moose locations with anthropogenic areas, we included two additional anthropogenic variables: the shortest distance from each locations to hard surface roads (referred to as roads) and to settlements (Topographic Objects Database) [47].

For each temporal subset, we ran seven conditional logistic regression models (“survival” package) [48]. In all seven models, the dependent variable was location type (observed – 1 vs random – 0), while the independent variables were the presence of specific habitat types: deciduous forests (mod1), coniferous forests (mod2), wetlands (mod3), grassland (mod4), arable land (mod5), and nearest distance to roads (mod6) and settlements (mod7). Models for anthropogenic areas, moorland, natural areas deprived of vegetation, and water bodies were omitted. To ensure straightforward interpretation of the model results, land use classes were represented as categorical dummy variables (1 – land use class of interest, 0 – other land use classes) [49] (Fig. 2). Before analysis, the road and settlement variables were standardized to a mean of 0 and an SD of 1. Step IDs were set as strata to account for data stratification (location pairs). In total, 4,368 models were fitted (624 subsets x 7 models).

From all 4,368 models, we extracted the model *β* coefficients to analyse the temporal patterns in moose habitat selection. For each habitat type, we ran generalized additive models with a Gaussian error structure using “mgcv” package (GAM1-7) [50]. The *β* coefficients served as the dependent variables, while time of day (hour), time of year (14-day long), and their interaction were used as explanatory variables, modelled with nonparametric splines [51] (Fig. 2). In all GAMs, we limited smoothing intensity to a maximum 10 knots for the time of year and 5 knots for time of day. We employed cyclic smoothers for both the time of year and time of day to account for the cyclical nature of these variables [51].

We tested temporal variation in summarized habitat selectivity of moose using a generalized additive model (GAM8). The response variable was the sum of absolute values of the *β* coefficients predicted by GAM1-5 for each day of the year (*n* = 365) and hour of the day (*n* = 24). We did not include the *β* coefficients predicted by GAM6 and 7 because they were on different scale and had a different interpretation compared to the coefficients from GAM1-5. Day of year and time of day, along with their interaction, were fitted as explanatory variables using splines with the approach used in GAM1-7.

Finally, we estimated the temporal repeatability in moose habitat selection. We correlated the habitat selection *β* coefficients of all analysed habitats (extracted from GAM1-7) across all 624 temporal subset (i.e. 14-day x 1-hour intervals), resulting in 194,376 pairwise correlations. An increasing correlation of habitat selection coefficients between pairs of temporal subsets indicated greater temporal repeatability in moose habitat selection. All habitat selection and statistical analyses were performed using R [52].

## Results

**Fig. 3:**
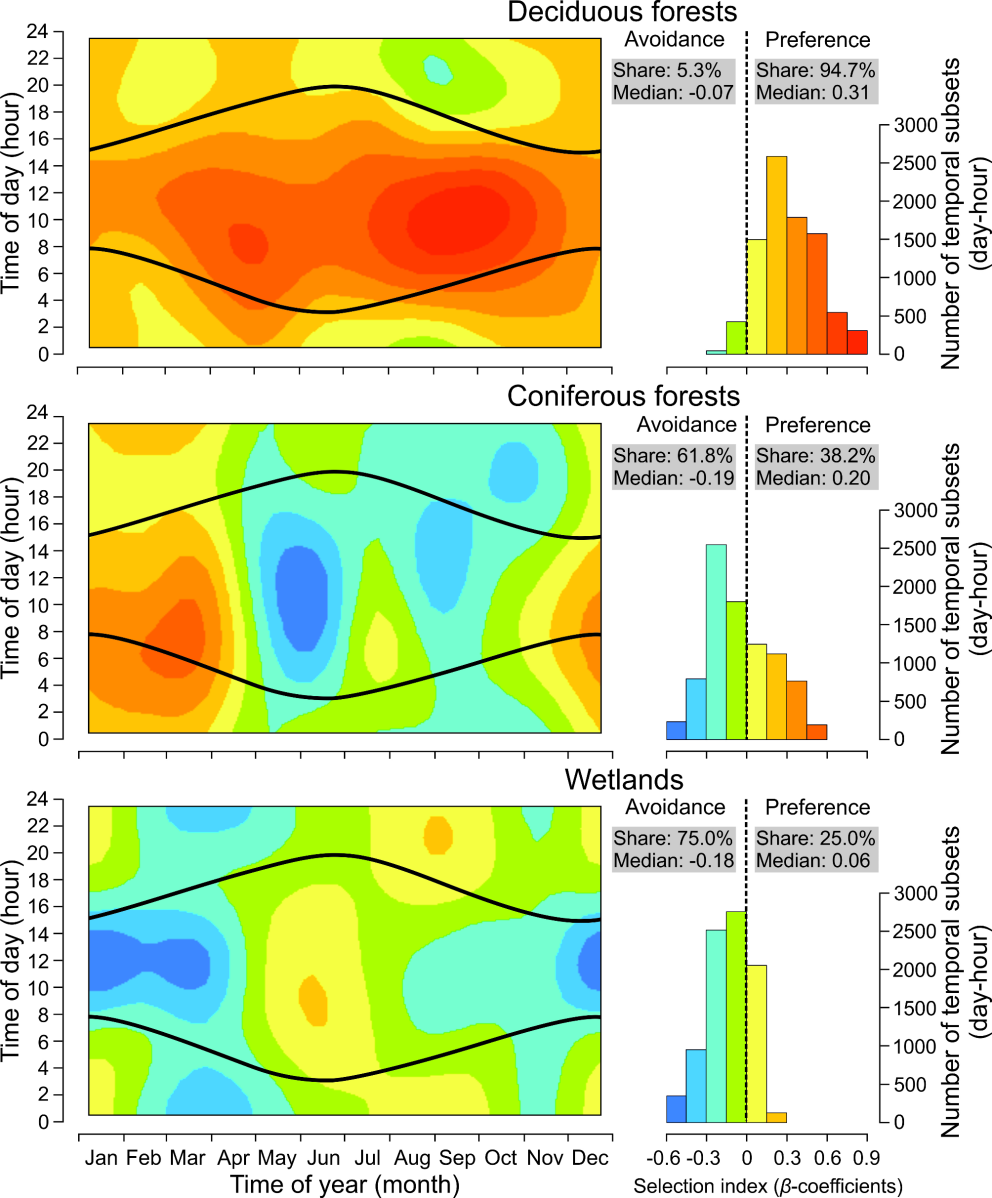
Predicted influence of time of year (1 January-31 December) and time of day (1–24 h) on habitat selection (*β*-coefficients) of natural habitats (deciduous forests, coniferous forests and wetlands) by moose in Eastern Poland (GAM1-3). Black curves indicate sunrise and sunset. Negative *β*-coefficients denote habitat avoidance, while positive *β*-coefficients indicate preference. The colours in the background of the heat maps refer to the colours in the histograms

The study revealed significant effects of time of day, time of year, and their interaction on moose habitat selection across all analysed habitats (GAM1-7, Table 1) indicating clear temporal patterns in habitat selection. For the majority of the time (94.7% of day-hour subsets), moose displayed a preference for deciduous forests. Within these day-hour subsets, moose were 1.36 (median of exp(*β*) – relative selection strength (RSS) [49, 53] times more likely to select deciduous forests over other habitat types (Fig. 3). Moose exhibited the highest preference for deciduous forests during daytime, with this preference remaining relatively stable across seasons. The strongest selectivity (RSS > 1.82) was observed between July and October, particularly from 06.00 to 14.00 h (Fig. 3). In contrast, moose avoided coniferous forests for 61.8% of the year, with the strongest avoidance occurring from May to October during daytime hours. However, moose displayed the highest selection for coniferous forests (RSS > 1.35) between December and March (Fig. 3). The preference and avoidance of coniferous forests showed a balanced pattern across moose’s temporal budget (day-hour subsets), with median RSS values of 0.83 for preference and 1.22 for avoidance. Moose exhibited low RSS values (below 1.18) for wetlands, with preference limited to the period between May and July. For most of the year, moose showed low, either positive or negative, selection for wetlands, being 0 to 1.35 times more or less likely to select wetlands than other habitat types. Moose strongly avoided wetlands (RSS < 0.74) between December and April (Fig. 3).

**Table 1:** List of summary information for fitted generalized additive models testing temporal pattern (daily and seasonal) in habitat selection by moose in Eastern Poland (GAM1-7). GAM8 described temporal pattern in a cumulative selection of habitats (sum of absolute values of *β* coefficients of all habitat considered except for distance to road and distance to settlements). *edf* – estimated degrees of freedom for the model terms; × – interaction between variables. For more details see Materials and methods section

Variable	Estimate ± SE or edf	t or F	*P*
*GAM1 – Deciduous forest*			
Intercept	0.31 *±* 0.01	45.3	< 0.001
Time of day	3.00	47.2	< 0.001
Time of year	6.65	2.40	< 0.001
Time of day × Time of year	15.2	10.1	< 0.001
*GAM2 – Coniferous forest*			
Intercept	-0.03 *±* 0.01	-3.49	< 0.001
Time of day	2.42	4.71	< 0.001
Time of year	8.00	22.5	< 0.001
Time of day × Time of year	14.0	4.11	< 0.001
*GAM3 – Wetland*			
Intercept	-0.13 *±* 0.02	-8.02	< 0.001
Time of day	2.63	1.59	0.02
Time of year	5.37	2.75	< 0.001
Time of day × Time of year	13.7	2.16	< 0.001
*GAM4 – Grassland*			
Intercept	-0.48 *±* 0.01	-45.3	< 0.001
Time of day	2.67	65.5	< 0.001
Time of year	7.73	8.28	< 0.001
Time of day × Time of year	13.2	4.00	< 0.001
*GAM5 – Arable land*			
Intercept	-0.63 *±* 0.02	-25.2	< 0.001
Time of day	2.77	25.7	< 0.001
Time of year	7.89	4.20	< 0.001
Time of day × Time of year	12.8	1.93	< 0.001
*GAM6 – Distance to road*			
Intercept	-0.08 *±* 0.02	-3.29	0.001
Time of day	2.22	8.60	< 0.001
Time of year	6.92	1.84	0.006
Time of day × Time of year	15.6	3.26	< 0.001
*GAM7 – Distance to settlement*			
Intercept	-0.19 *±* 0.04	-5.31	< 0.001
Time of day	1.71	5.19	< 0.001
Time of year	7.15	3.18	< 0.001
Time of day × Time of year	19.1	4.25	< 0.001
*GAM 8 – Cumulative selection of habitats*			
Intercept	1.89 *±* 0.001	1152	< 0.001
Time of day	3.00	27,290	< 0.001
Time of year	7.99	1084	< 0.001
Time of day × Time of year	24.0	681	< 0.001

**Fig. 4:**
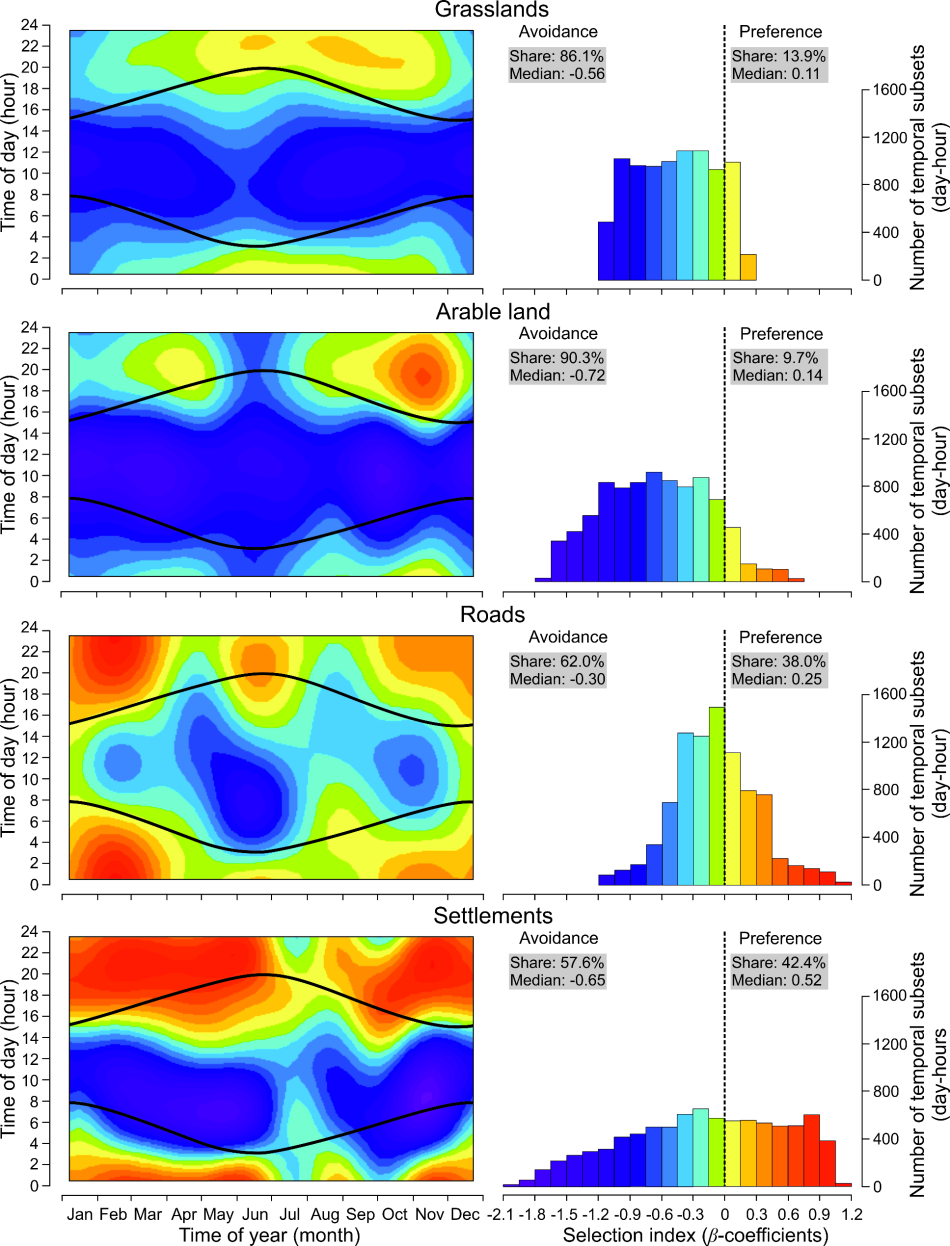
Predicted influence of time of year (1 January-31 December) and time of day (1–24 h) on habitat selection (*β*-coefficients) of anthropogenic habitats and objects (grasslands, arable land, roads, settlements) by moose in Eastern Poland (GAM4-7). Black curves indicate sunrise and sunset. Negative *β*-coefficients denote habitat avoidance, while positive *β*-coefficients indicate preference. The colours in the background of the heat maps refer to the colours in the histograms

For most of the temporal budget, moose avoided human-related habitats. Specifically, moose avoided grasslands for 86.1% of the available time (Fig. 4). When grasslands were negatively selected, the median RSS was 0.57, while during periods of positive selection, the median RSS was 1.12. The selection of grasslands by moose exhibited a significant diel pattern, with the strongest avoidance (RSS < 0.47) occurring during daytime hours (from 05.00 to 15.00 h; Fig. 4). Arable land was negatively selected by moose for 90.3% of the year, with a median RSS of 0.49 (Fig. 4). Regardless of time of year, the highest avoidance (RSS < 0.30) occurred during daytime hours (from 06.00 to 14.00 h), during which moose were at least 3.32 less likely to select arable land compared to other habitat types. However, moose showed a preference for arable land during autumn during nighttime hour, with this preference peaking twice – once in March-April and again in October-November, between 16.00 and 24.00 h – with higher selectivity observed in autumn. Roads exhibited mixed pattern of avoidance and preference over the year, with roads being avoided for 62.0% of time (RSS < 0.74). Avoidance was most prominent during daytime hours, with the greatest avoidance (RSS < 0.47) occurring from mid-April to mid-July between 04.00 and 16.00 h (Fig. 4). Conversely, roads were preferred by moose during nighttime hours in winter, with the highest preference (RSS > 1.57) occurring between January and mid-March from 18.00 to 05.00 h (Fig. 4). Moose spent approximately equal amounts of time avoiding and preferring settlements, with avoidance occurring 57.6% of the time and preference 42.4% of the time. The median RSS for avoidance was 0.52, while the median RSS for preference was 1.68. Over the year, moose primarily avoided settlements during daytime hours but were more likely to be closer to them during nighttime hours. This selection pattern weakened during the summer (July-August) (Fig. 4).

**Fig. 5:**
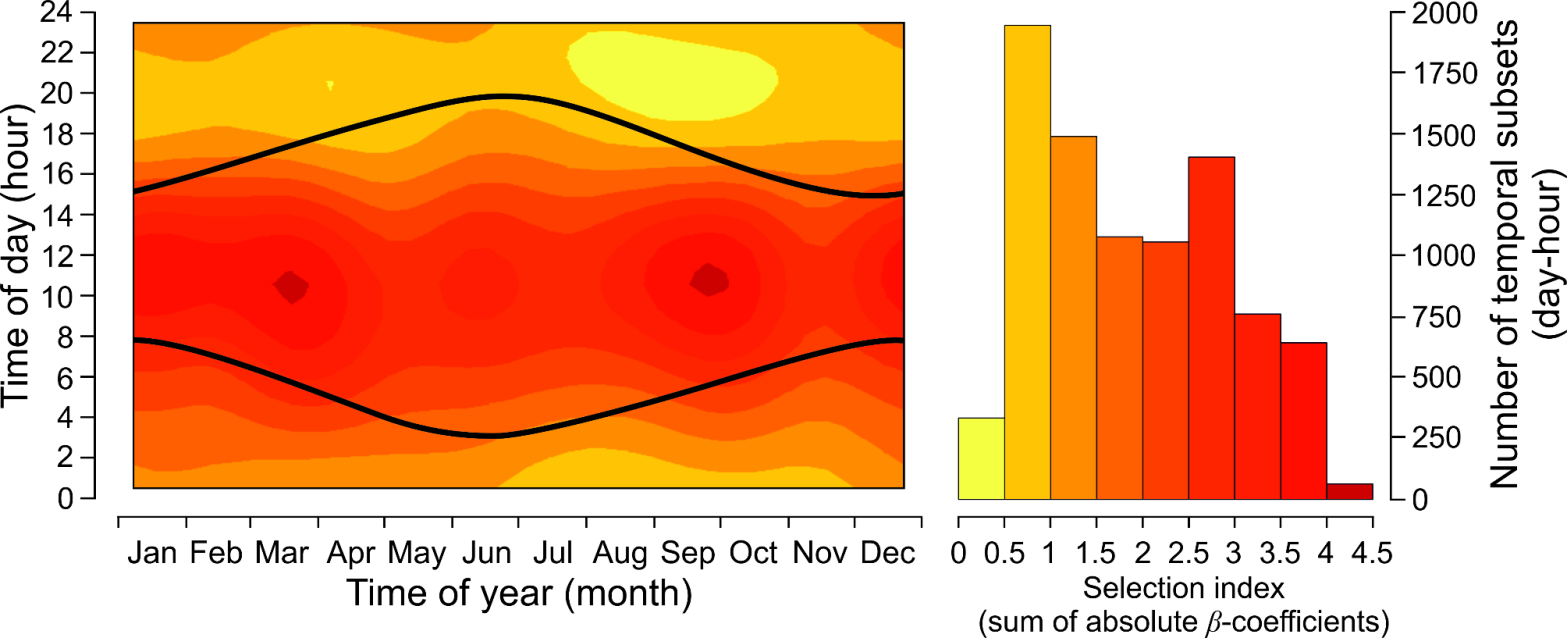
Predicted influence of time of year (1 January-31 December) and time of day (1–24 h) on the cumulative selectivity (sum of absolute *β*-coefficients) of habitats (deciduous forests, coniferous forests, wetlands, grasslands, arable land) by moose in Eastern Poland (GAM8). Black curves indicate sunrise and sunset. The colours in the background of the heat maps refer to the colours in the histograms

The results of GAM8, which summarized the temporal selectivity of moose toward considered habitat types (without roads and settlements), indicated that time of day, time of year, and their interaction were significantly related to the cumulative selection index. Moose exhibited greater selectivity during daytime hours compared to nighttime throughout the year (Fig. 5). There were two time windows of high selectivity (sum of absolute *β* above 3.5) between 8.00 and 13.00 h. These periods occurred from September to mid-October and from mid-December to mid-April (Fig. 5). Notably, diel changes in cumulative selectivity were more pronounced than seasonal changes, with a 5.6-fold difference compared to a 1.4-fold difference, respectively.

**Fig. 6:**
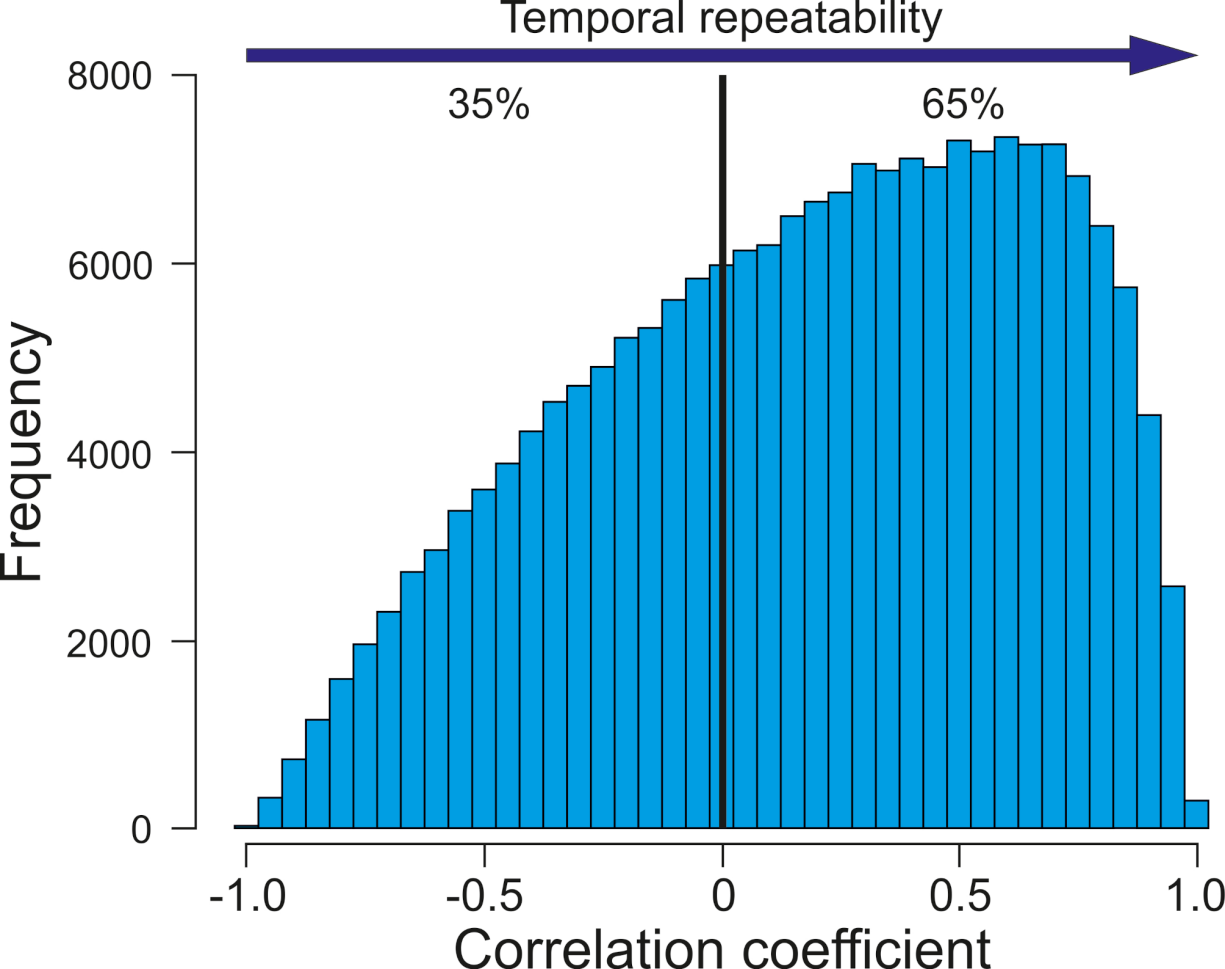
Temporal repeatability of habitat selection by moose. For more details see Materials and methods section

Finally, the similarity of habitat selection in moose across different temporal subsets was generally low. In 35% of pairwise comparisons between random subsets, the correlation was negative, indicating that habitat selection in one temporal subset was often opposite to that in another. In other words, habitats preferred in one temporal subset were avoided in another, and vice versa (Fig. 6).

## Discussion

Our study has shed light on the significant seasonal and diel variability in moose habitat selection within heterogeneous landscapes. Interestingly, these variations were highly habitat-specific. The selection of natural habitats primarily exhibited seasonal variation, while diel temporal variation, was more evident in human-altered habitats. These findings align with our predictions, which assumed that the daily pattern of habitat selection in anthropogenic habitats is primarily driven by human disturbance, whereas factors such as predator avoidance, food availability, and adverse weather conditions influence moose selectivity on a seasonal scale in natural habitats. Notably, cumulative habitat selection across all habitat types was clearly higher during daytime compared to nighttime, while seasonal differences were much less pronounced. The possible mechanisms driving these counterintuitive patterns and their ecological consequences are discussed below.

Moose consistently favoured forest habitats throughout the year, although the temporal patterns of selection differed between deciduous and coniferous forests. Deciduous forests were preferred year-round, particularly during the summer-autumn period, while coniferous forests were selected mainly in winter. These findings align with previous studies, which showed that the selection of different forest habitat types changes across various temporal scales, with a significant emphasis on the seasonal scale. In general, moose tended to select richer forest habitats in different seasons to meet their energy demands related to lactation (for females), body and antler growth (for males), and maintaining body condition [54–57]. However, our study found that the selection of rich forest habitats can also vary on a diel level. The significantly higher preference for deciduous forests during midday hours in the vegetation season may be related to the cover these habitats provide against human, predators, or high temperatures [19, 57–59]. Closed-canopy coniferous stands, on the other hand, were indicated as a crucial winter feeding habitat for moose worldwide, with their importance increasing during mid-winter and late winter [60]. Interestingly, we demonstrated that in spring, during one month (April), moose selection for coniferous forests shifted from clear preference to deep avoidance. This rapid change coincided with vegetation green-up and the moose migration from winter to summer ranges dominated by wetlands [29]. Finally, we found that wetlands were most attractive to moose during spring and early summer, when the animals occupied their summer ranges. The preference of wetlands in summer was likely linked to the high quality of forage and the presence of numerous water bodies, where moose could cool off or escape the harassment of biting insects [61]. Additionally, wetlands may be of great importance to female moose around the parturition period, as the location of calving sites is often associated with water availability during lactation period [62, 63]. It is also noteworthy that the significance of wetlands for moose gradually decreased from late summer, likely due to the deterioration of forage quality as a result of senescence processes [25, 26, 64].

In contrast to the seasonal variation in moose preferences of natural habitats, we showed that the temporal pattern in moose selection of anthropogenic habitats occurs primarily on a diel scale. Moose generally avoided human-related habitats during daytime hours when the probability of encountering humans is highest, suggesting that moose perceive these areas as high-risk [65]. However, we also observed differences in the selection of anthropogenic habitats at the seasonal level. A particularly striking example is arable land, which moose avoided throughout most of the year, except during spring and autumn nighttime hours, when it was clearly preferred. Notably, the importance of arable land for moose peaked in November, likely because winter crops provide higher quality forage at that time compared to other land use types [57]. Utilizing winter crops in autumn may represent a final opportunity for moose to improve their body condition before winter, when food resources become scarce. Interestingly, the temporal pattern of arable land selection appears to be site-specific. For instance, moose in Southern Sweden did not select for agriculture land during any season [66], whereas in the Canadian praire ecozone, moose selected crops equally in summer and winter, with different crop types being preferred at varying intensities [61]. In Norway, in turn, moose selected cultivated land predominantly at night, especially during the summer [65]. Our findings regarding grassland selection align with the widely reported avoidance of this habitat by moose [57]. Feeding on grass or hay can cause adverse digestive reactions or wasting syndrome complex in moose [67]. As a result, grasses constitute less than 1% of the moose diet and are mainly consumed in spring, when their digestibility is highest [68].

The most substantial daily differences in habitat selection occurred for areas located in the vicinity of road and settlements. Our result suggests, that moose clearly avoided these areas during daytime hours, most likely because of elevated risk related to human presence, but preferred them at night when human disturbance is much lower [69]. In case of roads, the greatest avoidance was indicated during spring and summer. This period coincides with time when females are with young at heel, hence most vulnerable to human disturbance [31, 69]. On the other hand, moose preferred areas near roads and settlements during nighttime hours which can be explained by the high attractiveness of forage in these places (ecotones) [70, 71]. Furthermore, moose in winter can visit roads to consume sodium from road deicing salt especially at night when traffic intensity is lower or moose can more frequently move along or on the roads where snow cover is usually thinner than in surrounding habitats [72, 73]. Areas near human settlements could be also selected by moose due to human-shield effects, i.e. animals might perceive these places as a zone of reduced predation risk [74, 75].

Finally, the greater variability in cumulative habitat selection on a diel scale compared to a seasonal scale suggests that factors following a diel rhythm, such as human disturbance, may have a more significant impact on habitat selection than those changing over longer temporal scales, such as plant phenology. This finding aligns with previous research by [9], which emphasized that avoiding human disturbance during daytime hours was the primary factor shaping habitat selection of red deer in predator-free, human-dominated environments. Although the variation in the strength of habitat selection on a seasonal scale was clearly weaker than the variation in diel rhythm, seasonal variation in habitat selection can still be of great importance. Multiple studies have evidenced that seasonal switches in habitat selection allow herbivores to adjust to temporal variations in food availability or escape predation and hunting mortality risk (e.g., domestic sheep (*Ovis aries*) [26], moose [76], roe deer [20]; woodland caribou (*Rangifer tarandus caribou*) [77]). Therefore, we do not underestimate the importance of seasonal differences in habitat selection in moose.

## Conclusions

To sum up, our study highlights the habitat-specific temporal variability in moose habitat selection within heterogeneous landscapes, which, in some cases, followed complex and non-linear patterns. This challenges the notion of discrete classifications into either seasonal or diel categories, which may be inappropriate, as they can mask real patterns and potentially underestimate the strength of habitat selection, leading to flawed conclusions. To better understand temporal variation in habitat selection, it is highly advisable to employ a continuous approach and examine the interactive effects of the considered temporal scales [11]. Moreover, habitat selection can change with time nonlinearly at any temporal scale, so applied models should be flexible and capable of capturing these curvilinear relationships [61]. The use of interaction of cyclic splines seems a reasonable option. Although not included in this study, interannual differences in habitat selection should be also considered, as weather conditions and vegetation properties (e.g., productivity and biomass) can vary significantly among years [78].

Since moose frequently come into conflicts with humans on one hand [31, 79], and are considered threatened in some regions of their distribution range on the other [80, 81], understanding their temporal habitat preferences is crucial. These preferences should be incorporated into both conservation programmes and management plans aimed at mitigating human-wildlife conflicts. In a broader ecological context, recognizing temporal variation in habitat selection by animals is of paramount importance, especially in the Anthropocene era, where a substantial proportion of habitats remain under human pressure and is impacted by changing climate [82]. Species may adapt to these changes not only by shifting their habitat selection (e.g., increasing preferences for less disturbed habitats) but also by altering their temporal patterns of habitat selection [83–85]. For instance, increased human disturbance may force shy species to select shelter habitats (e.g., dense vegetation) more frequently during the day [23, 86], while heatwaves could lead to avoidance of habitats with high insulation during the summer and daytime [19, 83]. While the overall selection of a certain habitat by a species may remain constant (i.e., constant share in its time budget), the temporal pattern of its selection may change dramatically, with significant consequences for the species’ ecology and fitness. Therefore, understanding the temporal regimes of habitat selection in wildlife requires high-resolution data, which can only be obtained through up-to-day survey methods (e.g., telemetry and camera-trapping). This knowledge is essential for effective conservation and management in a rapidly changing world.

## Data Availability

Data used in statistical analyses are stored in the Open Forest Data repository at https://doi.org/10.48370/OFD/UAMTSY.

## Abbreviations

GAM: generalized additive model
RSS: relative selection strength
